# “All of Me Is Completely Different”: Experiences and Consequences Among Victims of Technology-Assisted Child Sexual Abuse

**DOI:** 10.3389/fpsyg.2020.606218

**Published:** 2020-12-07

**Authors:** Malin Joleby, Carolina Lunde, Sara Landström, Linda S. Jonsson

**Affiliations:** ^1^Department of Psychology, University of Gothenburg, Göteborg, Sweden; ^2^Department of Social Sciences, Ersta Sköndal Bräcke University College, Stockholm, Sweden

**Keywords:** technology-assisted child sexual abuse, victim, internet, online abuse, thematic analysis, experiences, CSA, consequences

## Abstract

The aim of the present study was to gain a first-person perspective on the experiences of technology-assisted child sexual abuse (TA-CSA), and a deeper understanding of the way it may affect its victims. Seven young women (aged 17–24) with experience of TA-CSA before the age of 18 participated in individual in-depth interviews. The interviews were teller-focused with the aim of capturing the interviewee’s own story about how they made sense of their experiences over time, and what impact the victimization had on them in the short and long terms. Thematic analysis of the interviews revealed a broad range of abusive experiences that had profoundly impacted the individuals’ lives, health and self-concepts. Three dominant themes emerged from the analysis – *From thrilling to abusive*, *Negative effect on health and wellbeing*, and *A new self after the abuse*. *From thrilling to abusive* captures the wide range of experiences described, starting from the child’s own sexual curiosity to descriptions of having been manipulated or threatened into engaging in sexual activity, as well as the sometimes long and complex process of understanding the severity of one’s experiences. *Negative effect on health and wellbeing* describes the victimization’s comprehensive impact on the life and health of the participants, how they blamed themselves for what had happened, and the struggle of having to live with the constant fear of pictures from the abuse resurfacing. *A new self after the abuse* depicts how the victimization impacted the way participants viewed and thought about themselves in relation to others, and distorted their views of their bodies. The findings are discussed in relation to previous research on both offline CSA and TA-CSA, as well as theoretical and practical implications.

## Introduction

In recent years, child sexual abuse that is conducted through the means of internet has received increasing attention in the media following several large-scale cases involving many victims, and numerous countries worldwide have reported an increase in cases (Bentley et al., 2019; Swedish National Council for Crime Prevention, 2019). In a ground breaking sentencing in 2018, Sweden became the first country in the world to sentence a man for the rape of a child, for crimes that had been conducted solely online (B 11734-17). The man, while located in Sweden, had coerced children in the United Kingdom, the United States, and Canada into performing sexual acts on themselves. Evidently, this type of technology-assisted child sexual abuse (TA-CSA) is of global concern and an issue that requires attention. Following the increase in TA-CSA victims worldwide, there is a significant desire to learn more about the phenomenon of TA-CSA. So far, research has largely focused on offenders and offending behavior (e.g., Briggs et al., 2011; Webster et al., 2012; Kloess et al., 2014; Black et al., 2015; De Santisteban et al., 2018), with attention more recently turning to its potential consequences. Although the existing knowledge remains sparse, initial results indicate that the consequences of TA-CSA can be as severe and harmful as offline CSA (Whittle et al., 2013; Hamilton-Giachritsis et al., 2017; Jonsson et al., 2019; Joleby et al., 2020a). Adolescents with experience of TA-CSA (and no offline CSA) reported more trauma symptoms than a reference group, at least at the same level as adolescents with experiences of penetrative offline CSA (and no TA-CSA) (Jonsson et al., 2019). A wide range of psychological consequences similar to those reported among victims of offline CSA (e.g., psychological suffering, self-harming or suicidal behavior, sleeping problems, trust issues, impaired relationships, and difficulties at school) were identified in court documents regarding victims of TA-CSA (Joleby et al., 2020a). It has been suggested that the permanence of pictures of the abuse, which often exist in TA-CSA, potentially complicate the impact of abuse even further and can lead to increased feelings of self-blame (Hamilton-Giachritsis et al., 2017). This argument resonates with a study showing that knowledge of abuse pictures simply existing, or knowledge of them having been distributed, was related to higher levels of post-traumatic stress symptoms compared to being exposed to undocumented CSA (Jonsson and Svedin, 2017).

The existence of a relationship between TA-CSA victimization and psychological suffering is thus initially supported. In order to gain a deeper understanding of what this relationship looks like and which processes are behind it, this study uses in-depth interviews with victims of TA-CSA. The aim is to provide a rich first-person perspective on the experiences of TA-CSA and the way it may affect its victims. This understanding is vital in order to provide victims with sufficient support and help for coping with their experiences. Worryingly, there is a common assumption that TA-CSA is a less severe form of sexual abuse. Research has shown that professionals demonstrate a limited understanding of TA-CSA, may view it as less serious, and fail to prioritize its victims by minimizing their abusive experiences (Hamilton-Giachritsis et al., 2017, in press). Legally, these crimes are also considered less violating and result in lower penalties for the offenders. Despite the worldwide agreement that children should be protected from all forms of CSA (UNICEF, 1989; Council of Europe, 2007), 162 countries have failed to criminalize sexual grooming of children online *unless* the offender also attempts to organize an offline meeting with the child (International Centre for Missing and Exploited Children [ICMEC], 2017). This leaves children legally unprotected in many cases of online sexual abuse, as not all offenders aim for such a meeting (e.g., Briggs et al., 2011; Joleby et al., 2020b).

Online sexual victimization can take many forms, such as online dissemination of abuse pictures (Martin, 2015), sexual solicitation (Mitchell et al., 2001), online grooming (Whittle et al., 2013), and sextortion (Wolak and Finkelhor, 2016). The focus of this article is on TA-CSA in which children have been incited to engage in online sexual activity. Such activity can include sexual chat, generating sexual photos and/or videos, performing sexual acts live via webcam, or engaging in sexually humiliating activities online (Whittle et al., 2013; Kloess et al., 2019; Joleby et al., 2020a). With regard to the relationship between offender and victim, TA-CSA includes victims who may have perceived the offender as being a romantic partner, as well as victims who have experienced pressure and threats (Whittle et al., 2013; Wolak and Finkelhor, 2016; Joleby et al., 2020b), and no offline meeting is required for the situation to be considered TA-CSA.

This article aims to provide a deeper understanding of the experiences of TA-CSA and its potential consequences. More specifically, the aim is to understand how victims of TA-CSA have made sense of their experiences over time, and what impact the victimization had on them in the short and long terms. As this study was conducted in Sweden, a brief introduction to the Swedish legislation is necessary. In Sweden, the age of sexual consent is 15 years (Swedish Penal Code, paragraph 6). Consequently, all sexual acts involving children under the age of 15 are illegal. In accordance with the United Nations’ Convention on the Rights of the Child (UNICEF, 1989), individuals under the age of 18 are defined as children. Thus, some sexual acts (if they harm the child’s health and development or if the child is under the care of the offender) between adults and children between the age of 15 and 17 are also illegal (Swedish Penal Code, paragraph 6).

## Materials and Methods

### Recruitment

Recruitment to the present study took place between spring 2018 and spring 2020. Initially, the recruitment criteria were (i) having been subjected before the age of 18 to TA-CSA (ii) which had been reported to the police. However, due to recruitment difficulties, the second criterion was excluded after a few months. Recruitment efforts were all aimed at a Swedish audience and included (a) inviting lawyers (*n* ∼ 50) to send recruitment flyers about the project to their clients in recent legal cases of TA-CSA, (b) distributing recruitment flyers in waiting rooms at youth clinics, youth centers, child and youth psychiatric centers, psychologists’ receptions, and support organizations in several municipalities, and (c) distributing digital recruitment flyers on social media via support organizations, spokespersons engaged in issues of sexual abuse, and celebrities who are known to debate issues of sexual abuse. The flyer included the recruitment criteria, brief information about the focus of the interview (“hearing about your experiences and how your health has been before, during, and after the abuse”), and information about the right to anonymity. Flyers were aimed at both males and females. The recruitment efforts resulted in nine people (all female) getting in touch to receive more information about the study. One of them asked for information to forward to a friend, and one was under the age of 15 and did not want to ask her parents for consent (which is a requirement according to research ethics guidelines in Sweden), and was thus not allowed to participate in the study. The remaining seven were booked for interviews. Six of the interviewees found out about the study through the same celebrity (a female Swedish artist, author, and social media personality), and the seventh participant did not remember where she had found the information.

All interviews were conducted during summer 2019. The interviews took place at the interviewees’ choice of location, at either a library or a university in the chosen city, according to the participants’ requests. The first author – the only researcher who knew the identities of the participants – had no prior relationship with the participants and only met them on the one occasion of the interview. The participants did not receive any compensation for their participation (but were offered water and fruit during the interview).

### Interviews

The interviews were based on the teller-focused interview method (Hydén, 2014) due to its suitability for interviews about sensitive topics. This method endorses a dialectical way of thinking about the relationship between the interviewer and the interviewee, in that they are two partners with different tasks and responsibilities during the interview. The aim is to reduce the power imbalance between the two, and to provide a relationally safe space in which the informant feels safe to share his or her story. The method is oriented toward narration and resembles unstructured interviewing (Brinkmann, 2014) in that it uses open questions. Before starting the interview, the first author informed the participants that the aim of the interview was to let them tell their story. The interview used open invitations like “Tell me about yourself and who you are” and “Tell me about what happened,” and open questions like “What was your life like before the abuse?,” which were followed up with cued prompts (“You said X, please tell me more about that”), facilitating utterances (nodding, “Umm,” etc.), clarifying questions (“Do you mean Y?”), and relevant follow-up questions. The aim of the interview was to allow the participant’s story to develop naturally, but still make sure that all preplanned topics were covered (friends, school, mental health, and family, etc.). Most questions were broad or open (see full interview guide in Supplementary Materials), and covered the life of the participants before, during, and after the abuse.

The interviews lasted for 36 to 90 min, with an average interview time of 68 min (this is the time of the audio-recorded interview and thus exclude the pre-interview information and the completion of questionnaires). Before the interviews, the participants received information about their participation (that it is voluntary, that they will remain anonymous, that they have the right to withdraw their participation at any time, how the data would be stored, and reported), and filled out a consent form. After the interviews, the participants were asked to fill out the Rosenberg Self-Esteem Scale (Rosenberg, 1965), the Linköping Youth Life Event Scale (Nilsson et al., 2010), and a questionnaire specifying the type of online abuse they had been exposed to and its context. They were given the choice to either fill out the surveys on the spot, or to take them away and post their answers afterward (pre-stamped envelopes were provided). All participants except one chose to fill out the survey on the spot. On leaving, participants received a pamphlet with contact information for support organizations working with victims of sexual abuse.

### Analysis

The aim of the study was to gain a first-person perspective on the experiences of TA-CSA, and a deeper understanding of the way it may affect its victims. Thus, qualitative methods with an explorative and descriptive approach fit well. The study employed a qualitative design by using thematic analysis, which is “a method for identifying, analyzing and reporting patterns within data” (Braun and Clarke, 2006, p. 79). The data was analyzed inductively, which involves open-minded (theory-free) exploration of data (Braun and Clarke, 2013), and generates themes that are closely linked to the data without any theoretical constraint. The analysis focused on the surface meaning of the data, identifying themes on a semantic level.

The procedures recommended by Braun and Clarke (2006) to ensure a rigorous thematic analysis were undertaken. All interviews were conducted, audio-recorded, and transcribed verbatim by the first author, who thus had a high degree of familiarity with the data prior to the commencement of coding. In order not to disembody the participants, but instead create a fuller contextual understanding of their stories, the thematic analysis used a case-based approach in which each transcript was systematically coded separately.

Throughout the analysis, an iterative approach was employed by revisiting and adjusting existing codes, revising themes, and checking the fit with the original data (Braun and Clarke, 2013). In order to understand how the participants made sense of their experiences and how they were impacted by the TA-CSA, the interview covered the lives and psychological health of the participant before, during, and after the abuse. Therefore, it was crucial to view each initial code in the context of the whole story of the participant. A table was created for each participant to give an overview of all initial codes that were identified within that participant’s story before, during, and after the abuse. After the initial coding, each initial code was revisited and compared to other initial codes within that participant’s story, in order to look for relationships between them. To be transparent (Yardley, 2008), we clarify with an example. One participant had an initial code of “had a lot of friends” before the abuse and “almost no friends” after the abuse. After revisiting the original quotations, it became apparent that this change in social network was due to the participant no longer daring to trust anyone. This more general part of the participant’s story was assigned the initial theme “Trust issues.” Another participant had the initial codes “Easy to be retraumatized”, “People are naïve”, and “The world is evil”, which were merged into the initial theme “Can’t trust anyone.” After creating one table for each participant, the initial themes were compared across participants. The initial themes “Trust issues” and “Can’t trust anyone” (together with similar initial themes from other participants) were merged into the overarching final theme “Difficult to trust people.”

The coding and naming of themes for the first three transcripts were carried out by the first and last authors, who coded each transcript separately and then created initial themes through discussion. The remaining four transcripts were initially coded and analyzed by the first author, and the last author verified that all coding accurately represented the raw data through a process of reading and re-reading all transcripts. The structure and finalized themes were set by the first and last author together, and approved by the second and third author. The iterative process of repeated discussions between two authors during the analytic process ensured that the findings were credible and dependable. Extracts from the interviews were used as illustrative examples to support the analytical claims (Braun and Clarke, 2013).

### Ethics

This research project was granted full ethical approval by the Regional Ethical Review Board in (Linköping, Sweden). According to Swedish law on ethical review of research (Swedish law, 2003:460, Paragraph 18), participants above the age of 15 have the right to consent to participate in research. Thus, no consent from the guardian or legal next of kin is needed.

### Participants

This study involves interviews with seven young women who self-identified as victims of TA-CSA. They were aged between 7 and 13 at the first occasion of the online abuse, and between 17 and 24 at the time of the interviews. All participants had been subjected to TA-CSA, and many of them had also been sexually abused offline either before or after the TA-CSA. See Table 1 for a summary of the participants’ experiences and survey answers. To provide a context for the data (Levitt et al., 2018), a short description of each participant, based on how they described themselves and their experiences during the interviews, follows. All participants’ names have been changed to pseudonyms and all identifying features removed to protect their identities. All quotes in the manuscript have been slightly edited to facilitate understanding, and translated to English.

**TABLE 1:** Characteristics and survey-scores across participants.

Name^*a*^	Age at onset of abuse	Length of contact with offender/s	Age at interview	Number of online offenders	Online sexual abuse^*b*^	Experience of offline sexual abuse^*c*^	LYLES score nIPE/IPE/ACC^*d*^	Rosenberg self-esteem scale score
Anna	13	5 years	24	Numerous	Penetration, sex in exhange for money	Yes, i.a. by online offenders (sex in exchange for money)	2/6/4	Low (4/30)
Beatrice	12	2–4 weeks	19	One	Penetration	No info	5/1/1	Low (15/30)
Clara	13	3 years	17	One	Penetration	No info	3/1/3	Low (4/30)
Denise	11	1 month and 6–8 months	19	Two	Penetration	Yes	9/8/7	Low (12/30)
Emma	13	2 years	20	Numerous	Penetration	Yes, by one of the online offenders	4/No info/No info	Low (11/30)
Frida	7	7 years	22	Numerous	Penetration, sex in exchange for money	Yes	8/2/6	Low (10/30)
Gabriella	11	2 years	19	One	Sexual conversations	Yes	9/3/6	Low (14/30)

*^*a*^All names are fictitious. ^*b*^The most serious type of sexual act that the child was incited to perform of sexual conversation, sexual posing, masturbation, and penetration, as well as whether they had received money in exchange for sexual activity. ^*c*^The question regarding experiences of offline sexual abuse was not limited to before the age of 18, thus the offline sexual abuse may have occurred any time during the participant’s life. ^*d*^LYLES = Linköping Youth Life Experience Scale, 41-item questionnaire measuring potentially traumatic life events. nIPE, non-interpersonal event; IPE, interpersonal event; ACC, adverse childhood circumstances. The figures indicate the number of experiences reported for each category. For comparison, a community sample of 172 non-bullied Swedish females (age 15–20) reported on average 6.39 nIPE, 1.87 IPE, and 1.09 ACC (Nilsson et al., 2012).*

**Anna** was subjected to psychological abuse by her parents while growing up. She explained that school was the only place where everything was fine. She was involved in many after-school activities, and only had a few friends. At age 13, when Anna’s grandfather had recently died, she turned to the internet to find someone to talk to. An older man initially listened to and comforted her, but eventually demanded that she take off her clothes and engage in masturbation. This was the first abusive experience that Anna encountered online. As Anna grew older, she started engaging in online sex as a self-harming behavior, which eventually resulted in her also being paid for sexual activities offline.

**Beatrice** described her childhood as being great, with many friends to hang out with. When she was 12 years old, she engaged in online sexual activity with a boy she thought was her age. Two years later, the police contacted her after finding videos of her on the boy’s computer during an investigation involving 30 other girls. The boy turned out to be much older than he had claimed, and Beatrice found herself suddenly involved in a legal process.

**Clara** stated that she was happy growing up, and that her childhood was nothing out of the ordinary. At the age of 13, she fell in love with a boy in her school class. The boy took advantage of Clara’s feelings and managed to persuade her to send him a picture of her in her underwear and to engage in sexual activity while chatting with him online. Six months after this event, he threatened to disseminate the picture of her unless she sent him a nude picture within a few hours. Clara was scared, and did as he demanded. Despite this, the boy disseminated the nude picture of her to his friends at school.

**Denise** portrayed herself as a super-extrovert child who loved being the center of attention and had lots of friends that she enjoyed hanging out with. While growing up, she suffered from anxiety and was sexually abused offline by her mother’s new boyfriend. At the age of 11, Denise had online contact with a boy she thought was her age. One night, he opened up his webcam and Denise was shocked to see a grown man masturbating. This event really scared her. When Denise was 14, she met a girl online whom she fell in love with. Due to the previous event, she asked for pictures of the girl to make sure the girl existed. Denise and the girl chatted online daily for many months. Eventually, Denise found out that the girl was in fact a man, who used the naked pictures she had sent to blackmail her into sending more pictures.

**Emma** explained that she grew up in a loving and caring family, and described herself as a really “good girl.” When Emma was young, she had good self-confidence, but grew more insecure as she approached adolescence. When she was around 13 years old, men started contacting her online. Although Emma felt a little disgusted, she also enjoyed the attention and the men managed to persuade her to engage in online sexual activity by manipulating her with compliments and flattery. One of the men communicated with Emma daily and convinced her that “love had no age limit.” Emma eventually met with him offline and was raped by him. Four years after this event, Emma was contacted by the police who had found pictures and videos of her on the man’s computer during an investigation involving 70 other children.

**Frida** reported that she grew up with supportive parents, but that she was a shy and somewhat unsocial child who was teased at school. Frida described her young self as worrying and having a lot of anxiety, but online she could be anyone she wanted and therefore used the internet to find people to talk to. At 7 years of age, men started taking the initiative for webcam sex, and Frida – who was initially curious and appreciated the affirmation it gave her – was persuaded. During a period of about 6 years, Frida was repeatedly incited to engage in sexual activities with men online.

**Gabriella** described herself as being an energetic and exuberant person who loves to be seen and heard, and has a great need for attention. At around 11 years of age, Gabriella spent a lot of time on the computer playing games, and found it exciting to talk to new people. One day she received a message saying “I miss seeing you in underwear.” Although confident that she had never sent anyone pictures of herself, she was worried that the man had hacked her webcam and she started chatting with him online. The man, who claimed to be 19 years old, showered Gabriella with compliments and questions. The man managed to persuade her to engage in increasingly sexual conversations during their 2 years of contact, but she refused to send nude pictures even when he tried to force her. About a year after the contact had ended, Gabriella was shocked to learn that 68 other children – some of whom had been forced to engage in extreme and violent sexual behaviors – had reported the man to the police.

## Results

The thematic analysis resulted in three main themes – From thrilling to abusive, Negative effect on health and wellbeing, and A new self after the abuse – with two, three, and three subthemes, respectively – see Table 2. Each theme is presented below, supported by illustrative quotations and relevant contextual information. Please note that participants sometimes used diminishing and mitigating phraseology when talking about their abusive experiences. Nonetheless, when an adult engages in sexual activity with a child, it is always the adult’s responsibility. The participants self-identified as victims of TA-CSA, and according to Swedish legislation their experiences would be deemed to constitute sexual abuse.

**TABLE 2:** Result from the thematic analysis (Braun and Clarke, 2006) of interviews with seven victims of technology-assisted child sexual abuse (TA-CSA) about their experiences and how the victimization affected them. A brief description of each sub-theme is provided.

Themes	Brief descriptions
**From thrilling to abusive**	
Falling into the hands of the offender	All participants had in different ways been allured, manipulated, or forced into engaging in online sexual activities, although some participants expressed that they at the time believed they voluntarily engaged in the sexual activities.
Realizing the severity	Most participants had a different understanding of their abuse looking back at it, as they first in retrospect understood the full severity of what they had been exposed to.
**Negative effect on health and wellbeing**	
Everything collapsed	Despite having different backgrounds, different experiences, and having their abuse revealed in different ways, all participants described having been negatively affected by the TA-CSA. Many of them suffered consequences that largely had affected, and still did affect, their lives and wellbeing, even though several years had passed since the abuse ended.
Self-blaming	The self-blame that many participants described had several sources. From shame about having been sexually curious or for taking an active role in the sexual activities, to shame for not having been able to shield themselves from the online abuse.
Fear of pictures resurfacing	The reality of, or the fear for, pictures being disseminated caused additional stress and led the after-effects of the online abuse to continue long after the abuse ended. This affected the psychological well-being of the participants for a long time, and for some it also affected the decisions they took regarding their future careers.
**A new self after the abuse**	
Trying to make sense of who I am	Some participants struggled with understanding who they were after the abuse. They believed that the victimization had fundamentally altered them as persons, and thus created a discrepancy between who they really were, and what themselves and others thought of them.
Difficult to trust people	For some of the participants, the TA-CSA had created a distrust in people and a skepticism of the good in others. This could result in participants limiting themselves or the people they surrounded themselves with.
Distorted view of my body	The abuse affected most participant’s relationship to and feelings toward their bodies in several ways. From leading to difficulties to view or enjoy their bodies or sexualities, to a failure to listen to their own boundaries or bodily needs.

### From Thrilling to Abusive

The analysis of how the participants made sense of the abuse revealed that their view and understanding of their experiences had changed significantly over time. The participants had been enticed or lured in different ways into engaging in online sexual activities, and most of them saw their abuse in a different light in retrospect than they did at the time of the abuse. The results are presented in the two themes *Falling into the hands of the offender* and *Realizing the severity*.

#### Falling Into the Hands of the Offender

All participants had different stories about how they had come into contact with their offender(s), ranging from having their curiosity exploited to being exposed to outright threats. Most participants, however, described how they had been duped by the offender’s skills in manipulating them in different ways. Some participants described the excitement of having someone showing an interest in them and making them feel seen and heard: ‘It was a very sensitive age. And coming into contact with someone was really big, almost thrilling in a way. I was very excited that “Oh, someone wants to talk to *me*”.’ Several participants also explained their curiosity and excitement at engaging in something previously unexplored. Beatrice explains:

“It was super exciting! It was… well, about the time where you start to explore yourself and stuff. So it was really cool, but also… top secret. I knew that mom and dad absolutely could not know about it. So it was really exciting, but also extremely important that no-one would find out.”

A common theme in the interviews was that the contact initially gave the participants a self-esteem boost and the attention they longed for, which made them overstep their own boundaries. Emma explained how the flattery prevailed over her doubts: “Since this person had showed an interest in me and believed I was cute and hot and all of that, I kind of wanted to do it, to get this affirmation, all the time.” When she expressed regret or doubts about the sexual activities, the offender showered her with compliments and manipulated her into thinking that their relationship was genuine and that she could say no if she wanted to. Gabriella also described how she was initially duped by her offender, who became increasingly aggressive throughout their contact.

“I thought it was love, that he cared about me. But that was not really the case, he wanted something completely different. But in my world, I guess I was naïve and believed he wanted to be my friend, and that he cared about me. […] And it’s easy for an 11-year-old to be naïve.”

Several participants were exposed to explicit threats. Anna, who was extremely upset after her grandfather had died, went online to find someone to talk to. She started chatting with a 60-year-old man on a chat roulette site, whom she initially thought was just being kind to her.

“To begin with, he was very… considerate? He asked why I was crying and what had happened. […] I felt pretty safe with him, and he let me cry. And then he asked me to remove my sweater, and I said “No, I don’t want to,” and he said that if I didn’t do it he would stop talking to me. […] I think I felt, I really needed that, to be listened to. So I did as he said. And he continued to talk to me as if nothing had happened, as if it wasn’t weird. So I kind of forgot about it.”

The man, however, took advantage of Anna’s trust and took a screenshot of her. He then threatened to search out her IP address, find her parents, and send the picture to them unless she engaged in different sexual activities. The man had a hold on Anna:

‘I remember I was crying, but for each thing I did, he said “Now I have a picture of *that.*” […] I didn’t know how my parents would react. Would they be able to talk to me? Would they be able to look at me if they found out what I had done? So I just did as he said, and he masturbated on cam.’

Gabriella also received threats about having pictures of her uploaded to the internet. When Gabriella started to realize that there was something devious about the “boy” she had been chatting with online and tried blocking him, he became angry. Although she was aware that the man did not have any naked pictures of her, she nevertheless felt threatened:

“Okay, he does not have any pictures, but what happens if he writes something, or manipulates a photo. […] I thought that people I don’t know very well on Facebook might see it and not understand that it is photoshopped, that he had done something to the picture. People would think it was me. So I was pretty scared.”

Instead of having the threats directed only toward herself, Emma described how one man threatened to commit suicide if she did not comply with his demands. Emma explained her reaction: “I panicked. Like shit, what if, what have I done then? If someone finds this [the chat logs], they might think I have kind of murdered someone.” Evidently, the threats made by the offenders were extremely effective. Anna, however, explained that the threats would not have worked on her today: “He said he knew where I lived. Of course he didn’t, I mean *now* I understand that he couldn’t know. But I didn’t really understand that at the time.”

#### Realizing the Severity

Between four and 15 years had passed between the (first occasion of) TA-CSA and the interview. For most participants, the passing of time had changed their perspective, and when they looked back at their experiences now, they had come to the realization that the situation was not what they initially thought it was. For instance, sexual activities that at the time felt relatively positive and exciting could in retrospect be viewed differently. This changed view could be due to participants growing older and gaining perspective. Emma said: “You do not have the ability to judge the consequences of your actions when you’re thirteen, fourteen, fifteen. You don’t see the consequences, that what you are doing is wrong, until maybe afterward.” Frida initially believed that the sexual contact with men online offered her experiences she would otherwise miss out on, and she appreciated the gifts and money she received. She described communicating with other young girls who were engaged in similar behaviors, and that she was caught up in it. This made it difficult to have a balanced view of what was going on, and it was only afterward that she realized the men had taken advantage of her: “The thing is, at the time you don’t really consider what is going on […], but afterward you realize how sick it was.”

For other participants, the changed view of the abuse occurred when they found out that the person they had engaged in sexual activities with had lied about who they were. This could turn an activity that they had experienced as seemingly mutually consenting, with someone they cared about and who they thought cared about them, into an experience of betrayal and abuse. Gabriella, who had been chatting online for about 2 years with what she thought was a boy who was a few years older, was shocked to find out about his true identity.

‘It evoked quite a lot of feelings, because I felt, I think it was a slap to the face, like “Oh, *this* is what has happened,” kind of. To just get all this served at one time was really tough, […] to find out everything, his identity, all the other people that had been abused, and stuff like that.’

Some participants were abused by numerous different people over the course of several years. While they experienced many of the situations as abusive and coercive, it was only in retrospect that they understood the full extent of the consequences it would lead to. What at the time might have appeared to be their own choice, had in fact been them being manipulated and used by people online. Emma explained how she realized in hindsight that “this person was not feeling well” when she looked back at herself. “That’s when it started to catch up with me, that I started to understand what I had been exposed to, and everything that had happened. Yeah, everything caught up with me.” Several participants described how they felt repulsed when they thought back to what they had been incited to engage in. Anna explained: ‘I felt very disgusted by myself and by people in general. Both what I had been asked to do, and that I had been able to do it to myself.’

### Negative Effect on Health and Wellbeing

Although some participants took a while to realize the full extent of their experiences, all participants described how the TA-CSA had negatively affected their health and wellbeing in both the short and long terms. This is presented in the three themes *Everything collapsed*, *Self-blaming* and *Fear of pictures resurfacing*.

#### Everything Collapsed

All participants had different psychological baggage before the online abuse occurred, and they all described their upbringing and mental health differently. Beatrice, Clara, Emma, and Gabriella all said that their childhood prior to the online abuse was good and safe, and none of them mentioned any previous psychological problems. By contrast, Anna, Denise, and Frida described suffering prior to the online abuse. Nevertheless, it was clear that all the participants were negatively affected by the abuse specifically, regardless of their previous mental health. Anna, who grew up in a dysfunctional family, said:

“Well I believe I was feeling… yeah, yeah I was already feeling bad (laughs) before. Or I was… lonely. A bit sad. Thought everything was a bit hopeless. But afterward… it feels like everything became much more precarious. It wasn’t that I was completely naïve about people being mean, and such. But it went from me being comfortable with the feeling of sadness (laughs), to me being completely turned off, and I started dissociating quite a lot.”

Similarly, Denise mentioned that she had suffered from anxiety as a child, but that she still lived a functional life before the abuse changed everything:

‘I talked less to people, I was making less contact with people, I didn’t use social media. I turned off most things. I had extremely high absence from school, and the school was like “You will lose all your grades”. Everything just fell apart for me. I had almost no friends. I ate my own emotions and felt like crap. I was scared and tired of trying.’

For Denise, Anna, and Frida, who were already struggling for other reasons, the online abuse made everything collapse and they became suicidal. The other participants, who seemingly lived relatively untroubled lives before the abuse, also described different forms of psychological suffering. Beatrice described how she was feeling very well and had a great upbringing, but that this suddenly changed when she developed depression following the realization of her abuse 5 years ago:

“I haven’t been genuinely happy for a longer period since before the whole police interrogation thing. Of course, I have happy moments and have had them for several years, but it has been several years since I was feeling good and happy in general.”

Aside from general depressive symptoms, sleeping problems, and anxiety, feelings of shame could also negatively affect the participants’ wellbeing. Emma mentioned the profound effect shame had on her:

“It was all the shame. It has like, kind of stopped me. This, all this shame. I was so extremely ashamed. And that is kind of, shame is maybe the feeling that is most difficult to bear, because it really paralyzes you. It affected how I wanted to live.”

Some described that the initial abuse taught them to ignore their own will, which resulted in them developing self-harming behavior which allowed other people to take power over their bodies. Anna explained: “I just did what people told me to. And afterward, I couldn’t really understand what I was doing. I remember that. That I had anxiety about it, like why do I do this? Why can’t I just quit?” Frida also described the negative spiral of destructiveness that followed the online abuse:

“Between the ages of 12 and 14, I really noticed that I, kind of, it sounds so sick, but I felt that I kind of wanted to sell my body, thoughts like that. And I don’t know why I felt like that, it was more that I felt like a failure, I would never find someone, so I might as well just do this, and then I could kill myself. […] The contact with men online made me feel so bad, you kind of lose your self-esteem, and sense of reality.”

All participants experienced negative consequences due to the abuse, but the trauma appeared at different times and in different situations, as described in a previous subtheme (Realizing the severity). In contrast to Frida and Anna, who described the TA-CSA as having been disgusting or traumatic, some talked about the online sexual activities in more neutral or positive terms, and the real trauma seemed to have come afterward when they realized that they had been tricked or deceived, or when they realized that other people had knowledge of the abuse. Beatrice explained that the sexual activity she engaged in with what she thought was a boy her age was “nothing I really thought about. It happened and then I moved on, kind of. It was nothing I gave much attention to, it was nothing I kept thinking about. Until this legal process.” Being thrown into a legal process and becoming aware that the sexual conversations, photos, and videos she had shared were now being viewed by others was traumatic. After the first police interrogation, Beatrice described “three to four years of pure shame about myself.” It seemed as if a large part of the trauma could stem from the social shame of having a secret revealed, and not only the realization that one has been subjected to abuse. Similarly, Emma described her reaction to the first phone call from the police, in which they explained that they had found pictures and videos of her on a suspect’s computer:

‘And I was like “Okay what kind of pictures?” And they were like “I think you know what kind of pictures,” and I was like “Well okay yes I know.” And then the world fell apart, I was extremely sad, I panicked.’

For almost all participants, several years had passed since the online abuse ended, but nevertheless, many of the participants described thinking about it a lot, struggling with ongoing depression, undergoing psychiatric evaluations, only being able to go to university part-time, or being in the midst of recovery. However, despite still struggling, most participants described a positive development and most saw some kind of brightening on the horizon. Frida said that she had not been thinking about it a lot during recent years, and Beatrice explained that she had been able to let go of the feelings of shame and instead felt anger: “I am mad. I am extremely mad. […] and not only at this man, but… at society as a whole.” Denise described her psychological health as going up and down:

“Some days are horrible, and I don’t want to get out of bed. Other days I can go outside for a run. And just be out in nature, and take my long walks and feel safe in that. So it varies a lot. But… it feels like it’s getting better.”

It is important to highlight that one interview stood out from the rest. Despite describing that she had indeed been negatively affected by the abuse, Gabriella, who had been encouraged to engage in sexualized conversations, also stated that it had not affected her too much*:* “Of course, it might have shaped me, but not very much, not so that it has left deep traces on me, but perhaps to some extent.”

#### Self-Blaming

A common theme was to put the blame on themselves for what they had been subjected to. For some participants, this was due to them believing that their sexual curiosity was something taboo, and that the activities they engaged in were inappropriate. Emma explained that she kept her online contact a secret, “so in some way I kind of knew it was wrong,” and Frida expressed that her behavior was “abnormal.” For others, the self-blame stemmed from having been fooled. Due to having been misled online before, Denise asked for photos and an address to make sure that the girl she was chatting with online was who she claimed to be. When she realized that she had been deceived by a man despite these precautions, she berated herself:

“I felt that *I* was so very stupid for having done this, like how can you trust someone you don’t actually know whether you know? […] I felt so very mad at myself for doing it, and often felt that if something happens now, it’s still my fault to some extent. I’ve contributed to it, erm, so I guess I only have myself to blame.”

Emma said that she did not dare to tell her friends about the abuse she was subjected to, as it would reveal her own stupidity:

“I remember thinking that this would *never* have happened to them. It is so typically me. Because they are so good and perfect and all that. They would 100% understand that this was a pedophile you were talking to, but I’m stupid, and I didn’t understand that.”

Another factor for self-blame was that the sexual abuse was conducted online and that there was no physical meeting between the participants and their offenders. Some participants expressed that they should therefore have been able to shield themselves from the abuse, and that if they had just turned off their computers, “none of this would have happened.” Emma explained:

“Since I was behind a computer screen all the time, I kind of, afterward I thought I had myself to blame. […] It has led me to think that *I* have not been subjected to anything, but it is like I have subjected *myself* to it.”

Similarly, the participants had to take an active role in their abuse, for instance by following directions or demands from their offenders, or by playing along with their sexualized conversations. Frida was involved in a legal process, during which she had to listen to her conversations with the offender being read aloud in court. She described how she felt guilty when she realized that she had also been taking the initiative.

‘I knew it was wrong somehow [the sexual activities], but I listened to what he said. […] I’ve repressed this so much, that I’ve been a driving force in this, but when I read, when I sat in the courtroom and had it read to me, I got like a shock, like “shit,” “what the hell”.’

Most participants expressed initial self-blame, but as more time passed after the abuse, many came to realize that the guilt was not theirs but the offender’s. Despite this realization, some struggled to rid themselves of the feelings of shame. Gabriella said that she is aware in hindsight that it was not her fault, but nevertheless said:

‘I still feel an underlying sense of shame, even if I don’t want it to be like that. It’s still not something that I would like to talk about. […] It might be that this shame is still in me today.’

#### Fear of Pictures Resurfacing

One of the major causes of anxiety was the existence of pictures or videos of the abuse. Anna was confronted by a colleague who recognized her from videos online, and this caused her such extreme stress that she did not return to that job. It is important to emphasize that it was not only when pictures were disseminated that anxiety or distress occurred. Just the fear that someone could see the pictures or videos was enough to cause concern, and numerous participants described worrying about who had seen them. Several participants described the existence of pictures as something of a ticking bomb; “I always have to be prepared” and “this will eventually come back to me.” Clara explained how she deleted her whole Facebook profile because she was so worried that the picture would be posted there: “I was so scared that my parents would see, and I didn’t want proof that I had done this.” Denise described how she was “just sitting at home, always indoors, waiting for something to happen. […] I was scared to death.” The fear was also present out in public, as Emma exemplified:

‘Every time an older man, or a man, gives me more than a glance when I walk past, I automatically think “This person has seen these videos, this is a pedophile.” It’s kind of sick to think like that. I don’t do it as frequently anymore, but when I was younger I thought like that right away, and I kind of panicked.’

For Anna, this worry caused her to develop a fear of men, resulting in her not being able to buy groceries for 2 years if the cashier was a man. Furthermore, the knowledge that compromising pictures or videos might exist could have far-reaching consequences in some of the participants’ life decisions. Emma used to think that her future was ruined: “[…] because, if I was to succeed with anything, and in any way become a public person, these videos would resurface. And then the whole world would see.” Similarly, Anna described that she had always wanted to be an author. At one time she was nominated for a literature award and had a real chance of becoming an author, but she realized: “No, that will not happen. I cannot have my picture and my name linked to something that I am not sure what will happen to, that is public.” She also described feeling that nothing online ever really ceased to exist, “so of course I am worried, but (sighs)… I try not to think about it too much or else I would not be able to have a life (laughs).”

A few participants also expressed concerns that they had done something illegal when producing the pictures. When Beatrice was summoned to the police to be questioned, she was assigned a lawyer. This convinced her that it was her who had done something wrong. Anna said that she was concerned someone would report her to the police:

“I had read somewhere that it counted as producing child pornography if you uploaded pictures or videos of yourself if you were under 18, and I remember being scared about that as well, that someone would find the pictures and report me.”

Another traumatizing aspect of having the abuse documented was described by Beatrice and Emma, who had gone through legal processes in which their videos and chat logs were scrutinized. Since the videos were part of the evidence, Emma had to sit outside the courtroom knowing that everyone in court was watching videos of her touching herself.

‘It’s so surreal, and it’s so very humiliating having to walk back in and sit down in the chair, look someone in the eyes and confirm “Yes, that is me in the video.” So it has been extremely strenuous. Partly due to knowing that there are people out there that you don’t know, you don’t know who has these pictures or where they end up, but also, I don’t know, having to live with the facial expressions, the looks you received walking back into the courtroom knowing what they have just watched.’

### A New Self After the Abuse

In addition to the psychological consequences caused by the abuse, the interviews revealed that the victimization also affected some participants’ self-concept and how they acted. This is presented in the three themes *Trying to make sense of who I am*, *Difficult to trust people*, and *Distorted view of my body*.

#### Trying to Make Sense of who I Am

During the interviews, participants received the open invitation “Tell me about yourself and who you are.” The answers revealed that some participants struggled with their views of themselves and how this contrasted with the views others had of them, or how they themselves wanted to be seen. More specifically, Anna, Denise, and Emma all described themselves as being viewed as “good girls” and that their online victimization threatened that façade. Denise explains: “I was always portrayed as such a good child, because school went really well, and I always did well. And then this happens. That is not who I am. And all of a sudden I am bad.” They described the online victimization as something wrong or immoral that they themselves were responsible for. Not living up to the high expectations of them created an internal incongruity that made them question themselves. Anna explained how it was very difficult to cope with the discrepancy caused by the fact that “one side of me was subjected to abuse, and one side was functioning as usual.” Anna was very active in student union projects at school, had a job outside school, and had excellent grades. She explained: “Everyone viewed me as proper and high-functioning […] and that was the image of myself that I liked. I was scared that someone would find the pictures or videos and come to realize who I *really* was.”

Clara, who was abused by a boy at her school, described how the boy also manipulated her into treating her friends and brother badly. This had affected Clara’s self-esteem, since she now believed that she could not “know what is right and wrong in a relationship.” She described how the victimization became a sign of her character: “I have a very hard time accepting myself, like my personality above all. Because all I do is just wrong and mean and evil.” Likewise, Anna also questioned her own character. When she was younger, her self-hatred revolved around her looks, her geekiness, or things she was teased for at school. Now, her self-hatred evolved into questions like: “Am I a good person? Am I dangerous? Am I ruined? Am I broken? Things like that.” She continued:

‘I look at myself like an “*after*” now. Before, I used to view myself as a whole person, that was just what I was, but now I feel like I’m living in some kind of closing credits to something, and so I have to try to make something good out of it. As if all the important things have already happened.’

Some participants described how being lured into engaging in TA-CSA affected their self-esteem and self-confidence, and Denise described how she struggled with coming to terms with the fact that the abuse had made her a different person: “It’s tough to realize that… that it has affected you so much. More than you think. Because all of me is completely different.”

#### Difficult to Trust People

During the interviews, it became apparent that the online abuse had led some of the participants to lose trust in other people. This manifested itself in different ways, and affected their lives to different extents. For Gabriella, Beatrice, and Denise, it resulted in them being very careful online with private accounts and cautious about what kind of information they shared about themselves online. Gabriella said: “I don’t trust many people on the internet. It takes quite a lot for me to feel like, okay, this is not a fake person but someone who is decent and does not want to hurt me.” She described how she used to be scared of being abused again, but that it had also taught her to be less naïve and easy to fool, which she viewed as some kind of positive. Denise also expressed that the victimization had changed how she operated online:

‘I get so paranoid always. As soon as I receive a request to follow me online […] I ask my friends “Do you know this person? Do you know who it is?” […] I keep everything private. I don’t upload anything that I’m not 100 percent comfortable with. I use the block button frequently (laughs).’

Both Beatrice’s and Denise’s distrust in people online also spilled over into their life outside the internet and affected which people they surround themselves with. Beatrice explained that she has chosen friends that “she can really trust.” Similarly, Denise described having a hard time letting people into her life, and “didn’t dare to trust anyone else” after the abuse. Before the abuse, she described herself as being “the one everyone wanted to hang out with. And I wanted to be with everyone (laughs). I *loved* hanging out with people.” By contrast, she described her current social network as going “from having had everyone and anyone, I now have three real friends, and my family.” Denise portrayed how her worry had also changed her in more ways than minimizing her network.

“I used to be a very outgoing person. I was… I was always the center of attention. […] I loved to be seen. I loved to be heard, and I would put on talent shows and sing and dance and show off. […] Now I don’t want to be out around people. […] I find it very hard to be seen. I don’t want to be the center of attention. I’m terrified of attention.”

Anna, who had been sexually abused by many men over a period of several years, also struggled with trusting people. She said that she had lost her faith in society and that it was so easy to re-traumatize herself by just going online and confirming that the world was an evil place. This made it difficult for her to have any friends, and she described the isolation as the most challenging part:

“It’s difficult to explain to people why I don’t trust others. It feels like people in general have a very… naïve view of the world (laughs). And they think that people are kind and good, and that there are specific indicators of what a kind and good person is, and that they could never know anyone who would be able to… (sighs) hurt a child for instance. And that makes it difficult… erm, to relate to other people. I feel very abnormal… and I think that that is the hardest part, because I have had to come to terms with the fact that I might have a future without many people around me, and figure out how I can make that feel valuable.”

#### Distorted View of My Body

All the participants except Gabriella reported that their victimization had affected how they viewed their body or how they thought about their sexuality. Beatrice described how she used to think of her body as a tool before the abuse:

‘Before it happened, I didn’t reflect on my appearance, it was my body and I needed it to go to school and to do stuff. […] What I’m working on most these days is what I call “clothing anxiety.” Picking an outfit in the morning takes at least an hour, and half of the time it results in me not being able to leave.’

Denise talked about how she did not want to see herself naked and could not look at herself in the mirror without feeling ashamed. As a result, she used to cover up as much as possible:

“I wore XXXL in everything, because then you can’t tell what I look like. Eh, and I’ve always thought that if people don’t see what I look like, then I can’t be accused of contributing to whatever might happen.”

At the time of the interview, several years had passed since the online abuse ended. Denise described how things were slowly getting better, and that she was learning to dress more the way she wanted to:

“It feels fantastic to finally not only look at these things that you want to wear, but also to wear them and show them off. But also that I can undress in front of my partner […] and we can have sex. These steps have been tough.”

These extracts demonstrate how the abuse could lead to a distorted view of, and guilt about, one’s appearance and sexual desire. Denise explained that she used to be open about her sexuality, but that the abuse led her to feel that she did not want to and should not enjoy sex after what she had been through. With other participants, having been exposed to other people’s sexualization at a young age seemed to have blurred the boundaries around their own sexuality and the right to their bodies. For Clara, Emma, and Frida, the abuse caused them to overstep their own limits and lose track of their self-respect for their bodies. Clara described how the abuse made her think that “any guy can decide over my body in some way.” Emma explained how she did not feel like she owned her own sexuality because she had always been told what to do. This led her to agree to things that she did not really want to take part in online, and she also allowed guys at school to touch her body because “the attention I received from guys kind of meant more to me than how I was feeling.” Frida said that the online abuse was what caused her distorted view of sex, which led her to have a dysfunctional relationship with men and to develop feelings of not being worth anything.

Anna, who developed self-harming behavior in which adults paid her for sexual activities, described how all her abusive experiences had led her to shut off her body:

“I don’t think about it. At all. Most of the time, if it does not give me discomfort, umm… I forget that it exists, which means that I do not reflect on the fact… that it’s cold outside. I feel very little physical discomfort.”

## Discussion

This study’s findings, from in-depth interviews with seven young females who self-identified as victims of TA-CSA, build upon previous research showing that TA-CSA may have severe consequences (Hamilton-Giachritsis et al., 2017; Jonsson et al., 2019; Joleby et al., 2020a) and provide an insight into the underlying processes between TA-CSA victimization and psychological suffering. Despite the participants’ different experiences (from being abused by a single offender, to several years of repeated abuse by numerous offenders), they all provided detailed accounts of how their victimization had a negative effect on their health and wellbeing, not seldom of extremely serious proportions. The abuse impacted on several aspects of their lives, such as their relationships with others, their self-respect, and their ability to cope with everyday life. Research has shown CSA to be predictive of internalizing outcomes (Muniz et al., 2019), and many of the consequences described in the interviews match those that several decades of research on offline CSA have reported, namely general depressive symptoms, re-victimization, sexual problems, anxiety, poor self-esteem, and interpersonal problems (Paolucci et al., 2001; Maniglio, 2009).

The impact of the abuse could be both direct and delayed, depending on the participant’s understanding of the abusive situation and the time taken to realize its severity. Understanding that one’s experiences can be labeled as sexual abuse can be a long and complex process (Hjelen Stige et al., 2020), and the online element may add extra complexity as it allow offenders to hide their identity, leading the victims to believe that they were communicating with a peer. Thus, for some participants, the realization of abuse was not an inner insight, but occurred when police contacted them and revealed the true identity of the offender, which could be a shocking experience. TA-CSA offenders are, however, not always deceptive (Wolak and Finkelhor, 2013). In these cases, the manipulation and psychological grooming (Craven et al., 2006) that the victims were subjected to led them to gradually gain an understanding of the true nature of their experiences as they grew older and gained perspective. For the participants, the boundaries for when an experience was considered abuse could evidently be blurred. This can have implications for professionals meeting this victimized group. Therapists and support workers should consider what impact the involvement of technology might have, and should be aware of the possibility that young people may have difficulties understanding their experiences as abuse. For the same reason, law enforcement should be cautious when approaching children whom they suspect have been victims of TA-CSA, in order not to cause the victim any additional trauma in connection with the disclosure, as the child themselves may not be aware that they have been exploited.

Participants often expressed that they had initially been excited and sometimes part of inciting the sexual activity. Considering that sexual curiosity is a significant aspect of development (e.g., Kastbom et al., 2012), and that digital advances have led many young people to explore their sexuality online (Valkenburg and Peter, 2011; Anastassiou, 2017; Madigan et al., 2018), this initial excitement is not a deviating trait. Offenders taking advantage of children’s natural curiosity, however, seems to impact on the self-blame experienced by the victims, and constitutes an additional obstacle to realizing they had been exploited. The fact that the abuse took place online (“should have been able to turn off the computer”) and required the participants to be active in the acts (“felt like I subjected myself to the abuse”) led to further self-blame. Professionals have also noted that victims of TA-CSA are more often blamed by others and seen as participating in the abuse than victims of offline CSA (Hamilton-Giachritsis et al., 2017). Higher levels of self-blame among CSA victims have, in turn, been associated with increased psychological distress (Coffey et al., 1996). This illustrates the importance of professionals dealing with abused children working to counter feelings of blame (Hamilton-Giachritsis et al., 2017).

Another consequence of the abuse being conducted via digital technology was the constant fear of pictures resurfacing. This fear was one of the major causes of anxiety and impacted the lives of the participants in several ways, contributing to the long-term effects of the abuse. Always having to be prepared to be confronted and fearing one might be recognized out in public contributed to the never-ending aspect of the abuse, which has previously been reported among victims of online abuse images (Leonard, 2010). In sum, these findings lend support to the idea that some aspects of TA-CSA complicate the impact of the abuse (Hamilton-Giachritsis et al., 2017).

Consequences following CSA are known to vary widely among victims (Maniglio, 2009), which was also evident in this study. In line with research showing the cumulative negative effect of numerous traumatic experiences (Felitti et al., 2019), the participants who reported psychological suffering and trauma previous to the TA-CSA seemingly experienced the most severe impact following the abuse (e.g., suicidality). The participant with the least extensive abusive experience (being enticed to engage in sexual conversations but managing to refuse to send nude pictures, while the other participants had been incited to penetrate themselves) stood out from the group in that she only reported limited suffering. Whether this is due to the type of abuse she had been subjected to cannot be determined. It may instead be that some individuals are not severely affected by TA-CSA, as research on offline CSA has shown (Kendall-Tackett et al., 1993).

This study identified both immediate and long-lasting negative impacts on the psychological health of the participants following the TA-CSA. One way of explaining how and why CSA can result in both short- and long-term consequences is by using the four traumagenic dynamics model (Finkelhor and Browne, 1985). The model suggests that the experience of CSA changes the child’s cognitive and emotional orientation to the world, by distorting the child’s self-concept, world view, and affective capacities. Organizing the results from this study under the framework of this model (Finkelhor and Browne, 1985) can thus aid in understanding the findings. The model proposes four different dynamics (traumatic sexualization, betrayal, powerlessness, and stigmatization) that mediate the psychological outcome of CSA (Finkelhor and Browne, 1985). Each dynamic can be expressed in many different ways, and when analyzing the findings from this study through the lens of this model, all four dynamics can be identified. Firstly, traumatic sexualization refers to having one’s sexuality shaped in developmentally inappropriate ways. This can, for instance, lead to sexual problems, sexual re-victimization, and negative attitudes toward one’s sexuality and body (Finkelhor and Browne, 1985), all of which were expressed in the interviews. Secondly, betrayal refers to discovering that one has been manipulated by a trusted person, and can result in distrusting people and lead to isolation (Finkelhor and Browne, 1985). This was captured in the theme *Difficult to trust people*. Thirdly, powerlessness refers to the process in which one’s will, desire, and sense of efficacy are continually contravened (Finkelhor and Browne, 1985). Several participants expressed how they ignored their own will and blindly followed the demands of the offenders. According to the model, powerlessness can result in fear, anxiety, and re-victimization, which were also identified in the interviews. Fourthly, stigmatization refers to the negative connotations surrounding sexual victimization, such as shame, guilt, and badness, or that the activity was seen as taboo or deviant. This view can become incorporated into one’s self-image and, for instance, lead to feelings of guilt and shame, as well as self-destructive behavior and suicide attempts (Finkelhor and Browne, 1985), which were all described in the interviews. While this study in no way provided a full evaluation of the applicability of the four traumagenic dynamics model on TA-CSA, it is evident that the experiences and consequences of TA-CSA follow the same pattern as those for offline CSA.

Another way of explaining both the short- and long-term outcomes following CSA is through the potential damage to self-concept that may occur in response to victimization. Broadly, self-concept refers to the way individuals think about, evaluate, and perceive themselves (Baumeister, 1999), and one of its core aspects is self-esteem. Self-concept is proposed to have a mediating role between CSA and its negative outcomes on psychological health, and the relationship between CSA and negative self-concept and self-esteem has been theoretically and empirically explored (Kendall-Tackett et al., 1993; Stern et al., 1995; Turner et al., 2010; Cantón-Cortés et al., 2012; Lamoureux et al., 2012; Halvorsen et al., 2020). This study, in line with studies on offline CSA, showed that the abuse influenced the ways in which participants thought about and understood themselves, as well as how they thought others would perceive them. The time period during which the participants were subjected to TA-CSA is an important time in regards to sexual development (Diamond and Savin-Williams, 2009), fitting in and being accepted by peers concerns (Steinberg, 2011), and the development of a positive self-concept (Berger, 2018). Therefore, negative sexual experiences during this sensitive period may have particularly crucial implications. With regard to self-esteem, the Rosenberg Self-Esteem Scale (Rosenberg, 1965) is the most commonly used measuring tool, and individuals with a history of offline CSA report a higher frequency of low and medium self-esteem, relative to individuals without such a history (e.g., García et al., 2019). In line with this well-established finding, all participants in this study reported low levels of self-esteem, expressed both during the interviews and in the Rosenberg Self-Esteem Scale screening (Rosenberg, 1965) reported in Table 1. Again, the processes between TA-CSA and subsequent psychological suffering do not seem to be unique, but follow the same patterns as those of offline CSA.

### Limitations

First, this study’s results are based on a relatively small sample (seven young female victims of TA-CSA). The small number of participants is due to the sensitive nature of the topic and thus the extreme difficulty in accessing this population. The sample size was, however, appropriate for the method chosen.

Second, all participants were self-selected and self-identified as victims of TA-CSA. It is possible that this self-selection resulted in a biased sample, as victims who have experienced a more negative impact of abuse may have been more inclined to share their experiences by taking part in the study. The majority of the participants reported experiences of offline sexual abuse in addition to the TA-CSA (occurring either before or after the TA-CSA). The focus of the interviews was, however, the TA-CSA, and any information that was brought up regarding other traumatizing experiences or the impact of these has been omitted from the analysis.

Third, several years had passed between the (first) occasion of TA-CSA and the interview, which means that the participants’ stories are based on their retrospective recollections of their experiences. While this might have affected what they remembered and reported, it also provided a valuable insight into how the perception and impact of the abuse had developed over time.

## Conclusion

This study advanced our knowledge of how victims of TA-CSA make sense of their experiences, and provided an in-depth understanding of the ways in which TA-CSA can lead to psychological distress. The stories from seven victims of TA-CSA illustrated the ways in which they experienced that their victimization had profoundly impacted their lives and health, in both the short and long terms. Besides serious negative impacts on their mental health and relationships with other people, victims described how the victimization impacted their self-concept by altering the ways in which they viewed themselves in relation to others. Adding this all together, it is clear that many of the consequences and the processes between victimization and psychological suffering are similar to those of offline CSA. In addition, two factors appeared to add complexity to TA-CSA victimization, that is increased levels of self-blame due to the victims’ own participation in the abuse and the fact that it was conducted online, and the long-lasting fear of pictures or videos of the abuse resurfacing.

In sum, viewing these results in light of other initial research demonstrate the potential severity of TA-CSA, and indicates the need to challenge the assumption that it is a less severe form of sexual abuse.

## Manuscript Contributions

Given the recent increase in police reports regarding TA-CSA, there is a significant desire to learn more about the phenomenon. So far, research has mainly focused on offenders and offending behavior, with attention more recently turning to its potential consequences. While there seems to be a common assumption that TA-CSA is a less severe form of sexual abuse, initial research indicate that the consequences can be as severe and harmful as for offline CSA. The aim of this study was to provide a deeper understanding of how victims of TA-CSA make sense of their experiences and its potential consequences. Interviews with seven victims of TA-CSA revealed the victimizations comprehensive impact on the life and health of the participants, and the sometimes complex process of understanding the severity of their experiences. In sum, the present findings provided a first-person perspective on the experiences of TA-CSA and the potential processes between victimization and subsequent psychological suffering.

## Data Availability Statement

No datasets can be shared due to the sensitive nature of the data.

## Ethics Statement

This study involving human participants were reviewed and approved by the Regional Ethical Review Board in Linköping, Sweden. Written informed consent from the participants’ legal guardian/next of kin was not required to participate in this study in accordance with the national legislation and the institutional requirements.

## Author Contributions

MJ, CL, SL, and LJ designed the study. MJ conducted the interviews. MJ and LJ performed the thematic analysis. MJ created a draft of the manuscript, which was revised by CL, SL, and LJ. All authors contributed to the article and approved the submitted version.

## Conflict of Interest

The authors declare that the research was conducted in the absence of any commercial or financial relationships that could be construed as a potential conflict of interest. The handling editor declared a past supervisory role with one of the authors SL.
