# Supplementary material for: “All of Me Is Completely Different”: Experiences and Consequences Among Victims of Technology-Assisted Child Sexual Abuse
Source: Front Psychol. 2020 Dec 7;11:606218. doi: 10.3389/fpsyg.2020.606218 (PMC7750528; doi:10.3389/fpsyg.2020.606218)
Supplement: Supplementary file 1 [file Data_Sheet_1.PDF]

SUPPLEMENTARY MATERIAL:
Interview guide used in Joleby, Lunde, Landström & Jonsson (2020). The interview guide has been translated to English (the interviews were conducted in Swedish).

Interview guide: victim of TA-CSA

Introduction/orientation

Before starting the interview, I will tell you a bit about how this will go about. I have some questions for you, both about you as a person, about what you have been through, how you feel today, and how you have felt earlier in life. The idea is that I want to hear *your* story and *your* experiences and feelings. There are no right or wrong answers. Sometimes I might repeat a question or ask you to elaborate on or talk more about something you mention. If there is any question that you do not want to answer – that is okay. It is also okay if you feel like you want to end the interview at any time.

We will talk for about an hour, and I will audio record our talk. The reason for audio recording is so that I do not miss or forget what we are saying here today. No one except my colleagues and me will listen to the recording.

Do you have any questions for me before we start?

About you

I will start by asking you some questions about you

Tell me about yourself and who you are

- Leisure time
- Friends/network
- Occupation

We meet because you have been subjected to sexual abuse online. I will ask you some questions about that in a while, but first I want to know more about you. I want you to think back on your life when you were younger. What was your life like then – before the abuse?

- Childhood
- Family relations
- Friends/network
- School
- Leisure time
- Psychological health
- Was your psychological health different at any time? How come?
- Your online life

About the online abuse

Now I will ask you to tell me a little about what you have been exposed to on the internet.

Tell me about what happened

- How did the contact start?
- What happened next?
- Thinking back: what did you think about what happened?
- Thinking back: how did you feel about what happened?
- How did it affect you?
- Do you know if there are any pictures or videos of you?
- What are your thoughts on potential pictures/videos?
- Do you know if pictures/videos have been disseminated?
- Was there anything about the contact that was positive?

Disclosure

Did you tell anyone about the abuse

YES

- Who?
- When?
- What happened?
- How did it feel?
- What was your experience when the abuse was disclosed?

NO

- Why not?
- Did you want to/tried to?
- What happened?
- How did it feel?
- What would you have needed in order to tell anyone?

- Was there something in particular that made it difficult to tell?

About the legal process (if applicable)

Now we will talk about your contact with the police and judiciary

Tell me about your first contact with the police

- First contact?
- Did you understand the process?
- Investigation, police interview, trial – How was it?
- Positive experiences?
- Negative experiences? What was the most difficult part?
- What would you have wished to be different?
- How was your psychological health during this time?

Psychological healt after the abuse

Now I would like for you to tell me more about if and how the experiences have affected you.

Tell me about yourself and your health after the abuse

- Was there anything that changed?
- Friends
- Family relationships
- Relations
- Body
- What has been the toughest?
- How do you think this affected you?
- How do you view yourself today?
- Have you received any support?
- Did you wish to?
- How did it affect you that the abuse occurred online?
- How is your health today?
- What are your thoughts today about your experiences?

Closure/follow-up

I dont have any more questions. Is there something that I have not asked about that you want to tell me about?

Do you have any questions for me?

How did it feel talking to me today?
